# Symptoms of Emotional Disorders and Their Co-Occurrence with Adherence Levels in Individuals Aged 55 and Older with Chronic Diseases

**DOI:** 10.3390/jcm14186415

**Published:** 2025-09-11

**Authors:** Anna Polak-Szabela, Irena Wrońska, Mariola Głowacka

**Affiliations:** 1 Department of Geriatrics, Collegium Medicum in Bydgoszcz, Nicolaus Copernicus University in Toruń, 85-067 Bydgoszcz, Poland; anna.polak@cm.umk.pl; 2 Collegium Medicum, The Mazovian University in Plock, 09-402 Płock, Poland; i.wronska@mazowiecka.edu.pl

**Keywords:** depression, anxiety, comorbidity, medication adherence, chronic disease

## Abstract

**Background/Objectives**: Adherence to therapeutic recommendations is a key factor influencing treatment effectiveness, particularly in older adults with chronic diseases. Emotional disorders, such as depression and anxiety, may significantly affect adherence and overall health outcomes. The aim of this study was to analyze depressive symptoms and trait anxiety in individuals aged 55 years and older and to determine their association with adherence to therapeutic recommendations. **Methods**: The study included 2040 participants (1406 women and 634 men) aged 55 to 100 years (mean age: 65 years), all of whom had chronic diseases, most commonly cardiovascular and metabolic conditions. The sociodemographic variables analyzed were age, gender, and education level. Emotional functioning was assessed using the State-Trait Anxiety Inventory (STAI) and the Beck Depression Inventory (BDI). Therapeutic adherence, defined as the extent to which patients followed their treatment plan, was measured with the Adherence in Chronic Diseases Scale (ACDS). **Results**: ACDS scores ranged from 0 to 28 points, with a mean of 23.84. The majority of participants demonstrated moderate adherence (1149 individuals; 56.3%), followed by high adherence (593 individuals; 29.1%), while low adherence was observed in 298 participants (14.6%). Age and education level were not significantly correlated with adherence (*p* > 0.05). However, BDI scores showed a weak but statistically significant negative correlation with adherence (r = −0.185; *p* < 0.05). Similarly, STAI scores demonstrated a weak but significant negative correlation with adherence (r = −0.203; *p* < 0.05). In addition, BDI and STAI results were moderately correlated with each other (r = 0.453; *p* < 0.05). No significant differences in adherence were observed between men and women. **Conclusions**: In this large cohort of over 2000 Polish adults aged 55 years and older with chronic diseases, higher levels of depressive and anxiety symptoms were consistently associated with poorer adherence, with the co-occurrence of both disorders further amplifying this effect. These findings provide confirmatory evidence from an underrepresented Central European population with multimorbidity and underscore the need for systematic mental health screening and adherence-focused interventions in older patients.

## 1. Introduction

Collaboration with medical personnel in adhering to therapeutic recommendations is a crucial factor determining treatment effectiveness [1]. Adherence levels are influenced by multiple factors [2]. The presence and co-occurrence of mood disorder symptoms can significantly impact therapeutic success in patients with chronic diseases [3,4]. Numerous studies have shown that patients with somatic diseases are at an increased risk of developing mental disorders compared to physically healthy individuals [5]. The most common psychiatric comorbidities in this population are depression and anxiety disorders. Among patients with chronic somatic conditions, depression occurs two to three times more frequently than in the general population, particularly among older adults with multimorbidity [5,6,7]. Chronic diseases most often accompanied by depression include cardiovascular diseases, stroke, Parkinson’s disease, cancer, and HIV/AIDS [8,9,10,11,12,13]. The prevalence of anxiety disorders is also high in the geriatric population and among individuals with chronic diseases, with estimates reaching up to 50% [14,15]. Anxiety disorders frequently co-occur with depressive disorders and are present in 23–60.4% of individuals diagnosed with depression. Conversely, more than three-quarters of patients with depression experience significant levels of anxiety, while depressive disorders are reported in 13–60% of patients with anxiety disorders [16,17]. The coexistence of chronic diseases and affective disorders—mainly depression and anxiety—is associated with poorer prognosis, greater risk of disability and dependence, higher treatment costs, and reduced adherence to therapeutic recommendations [18]. Symptoms such as apathy, psychomotor retardation, reluctance to engage in conversation, negative outlook on the future, and inner restlessness may all contribute to poor cooperation with the therapeutic process in patients suffering from depression, anxiety, or mixed anxiety–depressive disorders.

Numerous studies have demonstrated that depressive and anxiety symptoms are associated with poorer medication adherence and reduced quality of life in patients with chronic diseases, including cardiovascular conditions. For instance, a systematic review of 31 studies reported that even mild to moderate depression significantly impairs treatment adherence in patients, e.g., with heart failure [19,20]. Poor mental health has also been linked to higher rehospitalization rates and diminished quality of life in individuals with chronic illnesses such as heart failure and diabetes [4,21]. However, many of these investigations were limited by relatively small sample sizes, a narrow focus on isolated psychological dimensions, or the lack of concurrent assessment of adherence, anxiety, depression, and quality of life. Moreover, comprehensive data from Central and Eastern European populations remain scarce [21,22,23].

The aim of this study was to examine symptoms of depression and trait anxiety in individuals aged 55 years and older and to assess their association with adherence to therapeutic recommendations.

## 2. Materials and Methods

### 2.1. Study Population

The prospective study included 2040 participants, of whom 68.9% (*n* = 1406) were women and 31.1% (*n* = 634) were men. Participants ranged in age from 55 to 100 years, with a mean age of 65.4 years. Eligibility criteria required individuals to be at least 55 years old, permanently reside or be registered in Płock, and be able to provide informed consent. Exclusion criteria included signs of dementia or refusal to provide consent.

Participants who enrolled in the study could complete the survey either in paper form or electronically via the dedicated LimeSurvey 6^®^ platform (GmbH, Hamburg, Germany). The online system required completion in a single session, recorded response times to ensure plausibility, and prevented multiple submissions from the same device or IP address. These safeguards ensured data validity and uniqueness.

Data were collected between January and November 2022 in primary healthcare facilities, patient homes, and Universities of the Third Age in Płock. A diagnostic survey and clinometric methods were applied.

### 2.2. Tools

Adherence scores were analyzed in relation to sociodemographic variables and measures of emotional functioning. The sociodemographic variables included age, sex, and education level. Emotional functioning was assessed using the Beck Depression Inventory (BDI) and the State-Trait Anxiety Inventory (STAI). Adherence, defined as the degree to which patients implemented their therapeutic plan, was evaluated with the Adherence in Chronic Diseases Scale (ACDS).

The State-Trait Anxiety Inventory (STAI) is a standardized tool adapted into Polish from the original American questionnaire. It enables the assessment of anxiety both as a transient emotional state and as a relatively stable personality trait. Raw scores range from 20 (low anxiety) to 80 (high anxiety), and are interpreted according to sten norms [24].

The Beck Depression Inventory (BDI) is a standardized tool used to assess the severity of depressive symptoms. It consists of 21 items, each rated on a scale from 0 to 3, reflecting the intensity of symptoms. Respondents select the statement that best describes their condition during the previous seven days. The total score is obtained by summing the responses across all items, with the following interpretation: 0–11 points—no depression; 12–19 points—mild depression; 20–25 points—moderate depression; and ≥26 points—severe depression [25].

The Adherence in Chronic Diseases Scale (ACDS) is a validated tool designed for adults with chronic conditions. It comprises seven items: the first five assess behaviors directly determining adherence (e.g., medication-taking behaviors), while the last two items capture situations and beliefs that may indirectly influence adherence, including aspects of the physician–patient relationship. The total score ranges from 0 to 28 points, with <21 points indicating low adherence, 21–26 points indicating moderate adherence, and >26 points indicating high adherence [26].

### 2.3. Statistical Analysis 

Parametric tests were applied to compare the obtained ACDS scores. Quantitative variables were presented using the arithmetic mean with standard deviation and confidence intervals, as well as the median and interquartile ranges. For comparisons of medians between two data series, the independent samples t-test was used (between groups). To assess relationships between quantitative variables, Pearson’s correlation coefficient was applied. The distribution of quantitative variables was assessed using the Shapiro–Wilk test. Due to deviations from normality and the large sample size, non-parametric tests were applied. A *p*-value of <0.05 was considered statistically significant.

## 3. Results

### 3.1. Study Characteristics

The study population comprised 2040 individuals, including 1406 women (mean age 64.8 ± 8.3 years) and 634 men (mean age 66.8 ± 8.0 years). The overall mean age was 65.4 years. More than half of the participants were between 60 and 75 years (52.6%), whereas only 1.2% were older than 90 years. With respect to education, 39.8% of respondents had secondary or post-secondary education and 27.8% had vocational education. Higher education was reported by 23.8%, while 8.6% had only primary education or less. Most participants reported at least one chronic condition. The most prevalent were hypertension (46.3%), musculoskeletal disorders (46.0%), and vision problems (44.0%), followed by urinary tract (30.8%) and pulmonary diseases (29.2%). Less frequent conditions included stroke (3.9%), chronic balance disorders (3.6%), tuberculosis (1.1%), AIDS (0.7%), and venereal diseases (0.5%).

### 3.2. Depression

Based on BDI scores, 69.3% of participants showed no depressive symptoms, while 3.9% presented severe symptoms. Women had slightly higher mean depression scores than men (8.9 vs. 7.9 points), although this difference was not statistically significant (*p* = 0.185). Severe depression was more frequent among women (4.6%) than men (2.4%). The full distribution of BDI categories is presented in Table 1.

Depression severity increased with age. A statistically significant but weak positive correlation was observed between age and depression levels (r = 0.237; *p* = 0.001). The highest mean depression scores were recorded in participants over 90 years of age (13.3 points) and in those aged 75–90 years (12.6 points), while the lowest scores were noted in individuals younger than 60 years (5.7 points). Detailed distributions across age groups are presented in Table 2.

Analysis of individual BDI items showed that symptoms most strongly contributing to overall depression scores were reduced sexual interest, fatigue, health-related worries, concerns about the future, difficulties in decision-making, and sleep disturbances. By contrast, the lowest mean scores were recorded for appetite loss, feelings of inferiority, weight loss, increased crying, and suicidal ideation. Detailed mean scores for all BDI items are provided in the Supplementary Materials (Table S1).

### 3.3. Anxiety

Most participants reported low levels of trait anxiety, whereas high anxiety was least common. The detailed distribution of STAI scores is shown in Table 3.

Women reported significantly higher trait anxiety levels than men (*p* < 0.05). The mean STAI score was 20.4 points in women and 19.0 points in men (Table 4).

Women were also more likely than men to score in the high trait anxiety range (14.6% vs. 9.9%; Table 5). In addition, age showed a weak but statistically significant positive correlation with trait anxiety levels (r = 0.078; *p* < 0.05).

Trait anxiety varied across age groups. The highest mean STAI scores were observed among participants aged 60–90 years (21.5 points), while the lowest were noted in those over 90 years (17.1 points). The proportion of individuals with high trait anxiety was also greatest in the 75–90 age group. A detailed age-based distribution is presented in Table 6.

Analysis of STAI trait anxiety items indicated that the highest mean scores were related to anticipatory fears (1.66), avoidance of uncomfortable situations (1.59), and physiological stress symptoms such as palpitations or stomach pain (1.55), while the lowest were linked to somatic restlessness (1.14), intrusive thoughts (1.03), and relaxation or calmness (≤0.9). Detailed item-level results are provided in the Supplementary Materials (Table S2).

Trait anxiety levels were significantly higher among individuals with depression. Half of the respondents with severe depression and nearly 40% of those with moderate depression scored in the high anxiety range, compared with only 5.6% among participants without depressive symptoms (Table 7). Overall, a statistically significant moderate correlation was observed between depression severity and trait anxiety intensity (r = 0.453; *p* < 0.001).

Most respondents demonstrated moderate adherence, while low adherence was relatively uncommon. No significant differences were observed between men and women (*p* = 0.199), and neither age nor education correlated with adherence (*p* = 0.497; *p* = 0.336). By contrast, depressive symptoms showed a weak but statistically significant negative correlation with adherence (r = −0.185; *p* < 0.05), indicating that greater depression severity was associated with poorer treatment adherence.

Mean adherence scores declined progressively with increasing depression severity. Participants without depressive symptoms had the highest adherence (24.2 points), whereas those with severe depression had the lowest (20.3 points). Similar patterns were observed for both direct adherence behaviors and indirect beliefs, with poorer scores consistently recorded among respondents with greater depressive symptomatology (Table S3).

High adherence was most common among respondents without depressive symptoms, whereas severe depression was associated with the lowest adherence; in this group, fewer than one in ten achieved high adherence, and low adherence was the most prevalent outcome (Table S4). Trait anxiety also correlated negatively with adherence (r = −0.203; *p* < 0.05), indicating that higher anxiety levels were linked to poorer treatment adherence.

Adherence declined with increasing trait anxiety. Respondents with low anxiety had the highest adherence scores, whereas those with high anxiety had the lowest. This pattern was consistent across both direct adherence behaviors and indirect beliefs. In the high-anxiety group, low adherence was the most frequent outcome, while in the low-anxiety group, high adherence predominated (Table S5).

## 4. Discussion

In the study group, the most frequent presentation of depressive symptoms was mild (20.7%), followed by moderate (6.0%) and severe (3.9%) cases. This distribution corresponds with previous findings in older adults, where subthreshold or subclinical depression with predominantly mild or moderate symptoms is most common [27].

No statistically significant differences were observed between men and women in terms of depression severity, which is consistent with earlier studies [28,29,30]. However, age was weakly correlated with depression severity, with higher scores recorded in older age groups. Comparable results were obtained in the PolSenior study, which reported that the prevalence of depressive disorders increased with age, reaching 33% among individuals over 80 years [31]. With respect to trait anxiety, the largest subgroup consisted of respondents with low anxiety levels (45.4%), whereas high levels were reported by 13.1% of participants. A statistically significant gender difference was observed, with women demonstrating higher trait anxiety levels than men. This finding aligns with prior research showing consistently greater prevalence of anxiety in women, regardless of age [15,32].

The co-occurrence of depressive and anxiety disorders is frequent in geriatric patients and significantly worsens prognosis, increasing the risk of chronic conditions and suicide. Depression is present in up to 60% of individuals with anxiety disorders [16], while 85% of patients with depression report significant anxiety [17]. In our study, depression scores showed a moderate correlation with anxiety levels (r = 0.453; *p* < 0.05), with higher anxiety observed in those with more severe depression. While this association is well established, our findings confirm it in a large population of pre-senior and senior individuals with chronic diseases and, importantly, demonstrate how this comorbidity affects adherence. In contrast, only 5.6% of participants without depression exhibited high anxiety scores. These results are consistent with prior research, which shows that anxiety in older adults is less often verbalized and more frequently expressed through worry, unfounded fears, cognitive deficits, or somatic complaints such as fatigue, pain, dyspnea, and tachycardia [33,34]. Such presentations complicate diagnosis and contribute to the underrecognition of depression in nearly half of geriatric patients [35]. Beyond statistical associations, psychosocial mechanisms may help explain the link between emotional disorders and poor adherence. Individuals with heightened emotional sensitivity may process health-related information differently, especially when it conveys negative emotional content. This may intensify worry, avoidance, or distrust regarding medical recommendations, thereby reducing adherence. Recent findings suggest that sensory processing sensitivity directs attention toward emotionally salient cues [36], which in older adults may interfere with the management of chronic disease regimens. This framework provides an additional explanation of how depressive and anxiety symptoms impair adherence, complementing the empirical associations demonstrated in our study.in our study.

Regarding adherence, most respondents demonstrated moderate adherence (56.3%), while 14.6% exhibited low adherence. Depression severity was negatively correlated with adherence, indicating that higher levels of depressive symptoms were associated with poorer adherence. The lowest adherence scores were recorded among patients with severe depression, of whom 42.5% demonstrated low adherence. These findings are consistent with prior research showing that individuals with depression are up to three times more likely to be non-adherent compared to those without depressive symptoms [37].

Similarly, trait anxiety levels correlated weakly but significantly with adherence (r = −0.203; *p* < 0.05). The highest adherence levels were found in individuals with low anxiety, while the lowest adherence was observed in those with high anxiety. These results align with studies showing that higher anxiety levels predict poorer adherence, particularly in patients undergoing hemodialysis [38] or cardiac rehabilitation [39] and those taking antihypertensive medication [40]. While the association between depression and anxiety is well established, our study extends previous knowledge by linking this comorbidity directly to treatment adherence in older adults with multimorbidity. Importantly, low adherence itself may worsen health outcomes, which can in turn exacerbate depression and anxiety, thereby creating a bidirectional relationship. This reciprocal influence has been highlighted in earlier research, which emphasized the mediating role of adherence in the relationship between depression and health outcomes [41].

Our findings are also relevant beyond gerontology and internal medicine, aligning with established concepts in health psychology and behavioral medicine. Depression and anxiety influence health-related behaviors through mechanisms such as reduced self-efficacy, increased avoidance, and altered illness perceptions, all of which may contribute to poorer adherence. Situating our results within this broader framework underscores that addressing mood disorders is not only a psychiatric or geriatric issue but also a central element of behavioral medicine approaches to chronic disease management. By framing adherence within a psychosocial model of health, our study contributes to the interdisciplinary understanding of how psychological factors shape health outcomes in older adults.

### 4.1. Practical Implications

The findings of this study underscore the need for integrated approaches to patient care, particularly for older adults with chronic conditions. Given the strong associations between depression, anxiety, and treatment adherence, healthcare providers should prioritize early screening and timely psychological interventions to improve adherence. Routine mental health assessments in both primary and specialized care may facilitate the early identification of high-risk patients and enable more effective intervention.

Multidisciplinary collaboration among physicians, psychologists, and rehabilitation specialists is essential for developing personalized treatment plans that address both medical and psychological needs. Training healthcare professionals to recognize psychosocial barriers to adherence, while incorporating behavioral strategies into chronic disease management programs, may contribute to improved long-term outcomes.

In addition, patient-centered educational initiatives that enhance mental health awareness and coping strategies could foster greater self-management and adherence to therapeutic regimens. Future research should investigate the effectiveness of tailored psychological interventions designed to strengthen adherence behaviors in patients with comorbid depression and anxiety.

### 4.2. Study Limitations

Several limitations should be acknowledged. First, the study relied on self-reported data, which may have introduced response bias. Second, voluntary participation could limit the generalizability of the findings, despite the high recruitment rate. Third, data collection took place during the COVID-19 pandemic; although restrictions were minimal at the time, it cannot be excluded that pandemic-related anxiety or uncertainty contributed to elevated levels of depressive and anxiety symptoms in some participants. Fourth, no subgroup analyses were performed to compare depression, anxiety, or adherence across specific chronic disease categories. The study was designed to capture these parameters in a large, heterogeneous population of older adults with multimorbidity, and the relatively small sample sizes in some disease groups would not allow for robust statistical comparisons. Finally, the cross-sectional design precludes causal inference. The observed associations between depression, anxiety, and adherence should therefore not be interpreted as directional. It remains equally plausible that poor adherence contributes to worse health outcomes, which in turn may secondarily increase depressive and anxiety symptoms. Longitudinal studies are needed to clarify the causal pathways underlying these relationships.

## 5. Conclusions

This study, conducted in a large cohort of over 2000 older adults with chronic diseases in Poland, confirmed significant associations between depressive and anxiety symptoms and treatment adherence. Greater symptom severity was consistently linked to poorer adherence, and the co-occurrence of depression and anxiety further amplified this effect. By demonstrating these associations in a Central European population of pre-senior and senior individuals with multimorbidity—an often underrepresented group—our findings extend the existing evidence base. The results highlight the importance of systematic screening for depression and anxiety in older patients with chronic conditions and emphasize the need for targeted interventions to improve adherence in this vulnerable population.

## Figures and Tables

**Table 1:** Depression levels in sex groups.

Parameter	All		Women		Men		*p*-Value
	*n*	%	*n*	%	*n*	%	
No depression	1414	69.3	969	68.9	445	70.2
Mild depression	423	20.7	291	20.7	132	20.8
Moderate depression	123	6.0	81	5.8	42	6.6
Severe depression	80	3.9	65	4.6	15	2.4
Total	2040	100.0	1406	100.0	634	100.0	0.185

**Table 2:** Depression levels measured using the BDI scale in age groups.

Age	Depression Levels	*n*	%	Mean BDI	SD	Median	*p*-Value
Under 60 years	No depression	551	83	5.7	6.83	3	0.001 *
	Mild depression	89	13.4				
	Moderate depression	12	1.8				
	Severe depression	12	1.8				
60–75 years	No depression	720	67.1	9.25	8.49	7	
	Mild depression	229	21.3				
	Moderate depression	80	7.5				
	Severe depression	44	4.1				
75–90 years	No depression	131	47	12.55	8.66	13	
	Mild depression	99	35.5				
	Moderate depression	27	9.7				
	Severe depression	22	7.9				
Over 90 years	No depression	12	50	13.29	13.31	10.5	
	Mild depression	6	25				
	Moderate depression	4	16.7				
	Severe depression	2	8.3				

* *p*-value based on chi-square test comparing distribution of depression severity across age groups.

**Table 3:** Trait anxiety levels in the STAI.

Anxiety Level	*n*	%
Low	927	45.4
Medium	845	41.4
High	268	13.1
Total	2040	100.0

**Table 4:** Differences in trait anxiety levels in the STAI between sexes.

Mean Score Woman	Mean Score Men	t	df	*p*	*n* (Valid)Woman	*n* (Valid)Men	SD Woman	SD MEN	Variance F	Variance *p*
1.706	1.614	2.775	2038	0.006	1406	634	0.707	0.661	1.145	0.049

**Table 5:** Trait anxiety levels by sex.

Sex	Woman		Men		*p*-Value
Anxiety Level	*n*	%	*n*	%	<0.05
Low	619	44.0	308	48.6	
Medium	582	41.4	263	41.5	
High	205	14.6	63	9.9	
Total	1406	100.0	634	100.0	

**Table 6:** Trait anxiety levels by age groups.

Age Group	Anxiety Level	*n*	%	Mean	SD	Median	*p*-Value
Under 60 years	Low	348	52.4	1916	862	20	<0.05
	Medium	238	35.8				
	High	78	11.7				
60–75 years	Low	459	42.8	2014	899	22	
	Medium	469	43.7				
	High	145	13.5				
75–90 years	Low	107	38.4	2152	804	23	
	Medium	130	46.6				
	High	42	15.1				
Over 90 years	Low	13	54.2	1708	945	195	
	Medium	8	33.3				
	High	3	12.5				

**Table 7:** Trait anxiety levels in depression scale groups.

Depression Level/Anxiety Level	No Depression		Mild Depression		Moderate Depression		Severe Depression		*p*-Value
results	***n***	%	***n***	%	***n***	%	***n***	%	
Low	840	59.4	69	16.3	9	7.3	9	11.3	
Anxiety	495	35.0	254	60.0	65	52.8	31	38.8	
High	79	5.6	100	23.6	49	39.8	40	50.0	
Total	1414	100.0	423	100.0	123	100.0	80	100.0	<0.001 *

* *p*-value refers to the correlation between depression severity (BDI) and trait anxiety intensity (STAI): r = 0.453; *p* < 0.001.

## Data Availability

The data presented in this study are available on request from the corresponding author. The data are not publicly available due to privacy and ethical restrictions.

## Supplementary Materials

The following supporting information can be downloaded at https://www.mdpi.com/article/10.3390/jcm14186415/s1, Table S1: The results of BDI; Table S2: Mean scores of State-Trait Anxiety Inventory (STAI) items; Table S3: Adherence scores in depression scale groups; Table S4: Adherence levels in depression scale groups; Table S5: Adherence in trait anxiety groups.

## Abbreviations

The following abbreviations are used in this manuscript:

ACDS	Adherence in Chronic Diseases Scale
AIDS	Acquired Immunodeficiency Syndrome
BDI	Beck Depression Inventory
COVID-19	Coronavirus Disease 2019
HIV	Human Immunodeficiency Virus
STAI	State-Trait Anxiety Inventory
