# Supplementary material for: Symptoms of Emotional Disorders and Their Co-Occurrence with Adherence Levels in Individuals Aged 55 and Older with Chronic Diseases
Source: J Clin Med. 2025 Sep 11;14(18):6415. doi: 10.3390/jcm14186415 (PMC12470817; doi:10.3390/jcm14186415)
Supplement: Supplementary file 1 [file jcm-14-06415-s001.zip › jcm-3812735-supplementary.pdf]

Suplementary materials

Table S1. The results of BDI

No.	Item	Median	SD	CI -95,0%	CI +95,0%	min	maks	Q25	median	Q75
1	Sadness and Pessimism	0,30	0,545	0,27	0,32	0,0	3,0	0,0	0,0	1,0
2	Concern for the Future	0,66	0,638	0,63	0,69	0,0	3,0	0,0	1,0	1,0
3	Self-Neglect	0,32	0,614	0,30	0,35	0,0	3,0	0,0	0,0	0,5
4	Loss of Pleasure	0,30	0,636	0,27	0,33	0,0	3,0	0,0	0,0	0,0
5	Feelings of Guilt	0,39	0,582	0,37	0,42	0,0	3,0	0,0	0,0	1,0
6	Self-Punishment	0,30	0,601	0,28	0,33	0,0	3,0	0,0	0,0	0,0
7	Self-Dissatisfaction	0,30	0,530	0,28	0,33	0,0	3,0	0,0	0,0	1,0
8	Feelings of Inferiority	0,25	0,514	0,23	0,28	0,0	3,0	0,0	0,0	0,0
9	Suicidal Thoughts	0,07	0,345	0,06	0,09	0,0	3,0	0,0	0,0	0,0
10	Increased Crying	0,20	0,559	0,18	0,23	0,0	3,0	0,0	0,0	0,0
11	Irritability and Nervousness	0,29	0,636	0,26	0,31	0,0	3,0	0,0	0,0	0,0
12	Loss of Interest in People	0,44	0,646	0,42	0,47	0,0	3,0	0,0	0,0	1,0
13	Difficulty Making Decisions	0,57	0,655	0,54	0,60	0,0	3,0	0,0	0,0	1,0
14	Self-Perception of Appearance	0,49	0,735	0,46	0,52	0,0	3,0	0,0	0,0	1,0
15	Reduced Work Performance	0,47	0,646	0,44	0,50	0,0	3,0	0,0	0,0	1,0
16	Sleep Disturbances	0,56	0,715	0,53	0,59	0,0	3,0	0,0	0,0	1,0
17	Fatigue and Loss of Energy	0,68	0,625	0,66	0,71	0,0	3,0	0,0	1,0	1,0
18	Changes in Appetite	0,25	0,530	0,23	0,28	0,0	3,0	0,0	0,0	0,0

19	Weight Loss	0,20	0,579	0,17	0,22	0,0	3,0	0,0	0,0	0,0
20	Health-Related Worries	0,66	0,915	0,62	0,70	0,0	3,0	0,0	0,0	2,0
21	Loss of Sexual Interest	0,86	0,984	0,82	0,90	0,0	3,0	0,0	1,0	1,0

Table S2. Mean Scores of State-Trait Anxiety Inventory (STAI) Items

No.	Item	Mean	SD	CI -95,0%	CI +95,0%	min	maks	Q25	median	Q75
1	I feel more nervous in certain situations than others	1,50	0,808	1,47	1,54	0,0	3,0	1,0	2,0	2,0
2	I experience muscle tension	1,30	0,869	1,27	1,34	0,0	3,0	1,0	1,0	2,0
3	In stressful situations, I feel physical symptoms such as rapid heartbeat, increased breathing, sweating, or stomach pain	1,55	0,862	1,52	1,59	0,0	3,0	1,0	2,0	2,0
4	I fear failure	1,54	0,886	1,51	1,58	0,0	3,0	1,0	2,0	2,0
5	I fear that something bad might happen to me	1,46	0,943	1,42	1,50	0,0	3,0	1,0	2,0	2,0
6	I have trouble falling asleep	1,39	0,968	1,35	1,43	0,0	3,0	1,0	1,0	2,0
7	I feel more sensitive than others	1,48	0,976	1,44	1,52	0,0	3,0	1,0	2,0	2,0
8	I have recurring thoughts that I cannot get rid of	1,03	0,942	0,99	1,07	0,0	3,0	0,0	1,0	2,0
9	I am calm and composed	0,78	0,816	0,75	0,82	0,0	3,0	0,0	1,0	1,0
10	Relationships with others cause me worry and anxiety	1,15	0,930	1,11	1,19	0,0	3,0	0,0	1,0	2,0
11	I relax easily	0,86	0,814	0,83	0,90	0,0	3,0	0,0	1,0	1,0
12	In stressful situations, my hands shake, or I feel a strong need to move my legs or walk	1,14	0,919	1,10	1,18	0,0	3,0	0,0	1,0	2,0
13	I tend to avoid certain situations in which I feel uncomfortable	1,59	0,895	1,55	1,62	0,0	3,0	1,0	2,0	2,0
14	I believe I react more strongly to some situations than others	1,53	0,916	1,49	1,57	0,0	3,0	1,0	2,0	2,0

15	I fear upcoming events	1,66	1,051	1,61	1,70	0,0	3,0	1,0	2,0	3,0

Table S3. Adherence Scores in Depression Scale Groups

Adherence	ACDS			p-value
BDI	Mean	SD	Me	
No Depression	24,16	3,96	25,00	
Mild Depression	23,56	3,31	24,00	
Moderate Depression	23,44	3,52	24,00	<0.05*
Severe Depression	20,26	5,85	22,00	

*p-value refers to correlation between BDI and ACDS: r = -0.185; p < 0.05

Table S4. Adherence Levels in Depression Scale Groups

Depresion	No Depression		Mild Depression		Moderate Depression		Severe Depression		p-value
ACDS	N	%	N	%	N	%	N	%	
Low	177	12,5	65	15,4	22	17,9	34	42,5	
Moderate	759	53,7	276	65,2	74	60,2	40	50,0	
High	478	33,8	82	19,4	27	22,0	6	7,5	
Total	1414	100,0	423	100,0	123	100,0	80	100,0	< 0.05*

*p-value refers to correlation between STAI and ACDS: r = -0.131; p < 0.05.

Table S5. Adherence in Trait Anxiety Groups

Trait Anxiety	Adherence Level	n	%	Mean	SD	Median	p-value
Low	Low	109	11,8	18,08	2,82	19	< 0.001*
	Moderate	431	46,5	6,56	1,6	7	
	High	387	41,7	24,64	3,98	26	
Moderate	Low	131	15,5	17,1	2,82	18	
	Moderate	563	66,6	6,2	1,44	6	
	High	151	17,9	23,29	3,74	24	
High	Low	58	21,6	16,74	2,94	17	
	Moderate	155	57,8	6,07	1,67	6	
	High	55	20,5	22,82	4,17	23	

* All correlations between BDI and WHOQOL domains were statistically significant: $p < 0.001$
